# Awareness and Knowledge of Hypothyroidism Among Female Health Science Students at Aljouf University: A Cross‐Sectional Analysis

**DOI:** 10.1155/nrp/4056149

**Published:** 2026-04-15

**Authors:** Shatha Basheer Aldababseh, Rabab Gad Abd El-Kader, Amal Ahmed Elbilgahy, Gehan Elnabawy Ahmed Moawad, Reham Elsaeed Hashad, Zeinab A. Ali, Ahmad M. Abdel-Mageed, Shereen Ahmed Elwasefy

**Affiliations:** ^1^ Department of Nursing, Zarqa New Governmental Hospital, Zarqa, Jordan; ^2^ Department of Community Health Nursing, Faculty of Nursing, Mansoura University, Mansoura, Egypt, mans.edu.eg; ^3^ Department of Community Health Nursing, RAK College of Nursing, RAK Medical & Health Sciences University, Ras Al Khaimah, United Arab Emirates, rakmhsu.com; ^4^ Maternal & Child Health Nursing Department, Faculty of Nursing, Northern Border University, Arar, Saudi Arabia, nbu.edu.sa; ^5^ Pediatric Nursing Department, Faculty of Nursing, Mansoura University, Mansoura, Egypt, mans.edu.eg; ^6^ Department of Pediatric Nursing, Nursing College, King Khalid University, Muhayil Asir, Saudi Arabia, kku.edu.sa; ^7^ Department of Pediatric Nursing, The National University of Science and Technology, Nasiriyah, Iraq; ^8^ Department of Physical Therapy and Health Rehabilitation, College of Medical Applied Science, Jouf University, Sakaka, Saudi Arabia, ju.edu.sa; ^9^ Department of Physical Therapy for Surgery, Faculty of Physical Therapy, Cairo University, Giza, Egypt, cu.edu.eg; ^10^ Department of Biological Sciences, College of Science, Northern Border University, Arar, Saudi Arabia, nbu.edu.sa; ^11^ Department of Nursing, College of Medical Applied Science, Jouf University, Sakaka, Saudi Arabia, ju.edu.sa

**Keywords:** awareness, female, hypothyroidism, knowledge, university students

## Abstract

**Background:**

Hypothyroidism is a prevalent endocrine disorder with potentially serious health implications if not diagnosed and managed early. Awareness and understanding of the condition are critical, particularly among future healthcare providers.

**Aim:**

The study was carried out to evaluate the extent of knowledge and awareness regarding hypothyroidism among female health science students at Aljouf University, Saudi Arabia.

**Subject and Method:**

A descriptive cross‐sectional study was conducted using a validated, self‐administered electronic questionnaire. The sample comprised 384 female students from various academic levels and specializations.

**Results:**

Nearly half of the participants (46.3%) demonstrated poor knowledge, while only 19% achieved a high knowledge level. Statistically significant associations were found between knowledge levels and age, academic level, field of study, and personal or family history of thyroid disorders (*p* < 0.001).

**Conclusion:**

The study highlights a substantial deficiency in hypothyroidism‐related knowledge among female university students. Recommendations: These results underscore the urgent need for targeted health education initiatives to improve thyroid health literacy among students, especially those not enrolled in nursing or health‐specialized programs.

## 1. Introduction

The thyroid gland plays a vital role in regulating metabolism, supporting physiological development, and maintaining systemic homeostasis. Among the various thyroid disorders, hypothyroidism characterized by insufficient secretion of thyroid hormones is one of the most prevalent endocrine conditions worldwide. Women are disproportionately affected, and the condition can manifest in either a primary form, due to intrinsic thyroid dysfunction, or a secondary form, resulting from extrathyroidal causes [1]. Clinically, the condition manifests across multiple organ systems, with symptoms including fatigue, weight fluctuations, cold intolerance, bradycardia, gastrointestinal disturbances, menstrual irregularities, and cognitive deficits. These symptoms often overlap with other diseases, complicating accurate and timely diagnosis. Measurement of serum thyroid‐stimulating hormone (TSH) and thyroid hormone levels remains the gold standard for diagnosis [2–5].

Global evidence highlights inadequate public awareness regarding thyroid dysfunctions, which contributes to delayed diagnosis and suboptimal disease management. Young women are vulnerable due to the implications of thyroid imbalances on reproductive and psychological health [6]. Awareness during reproductive years is especially vital to safeguard maternal and fetal outcomes [7]. Within the Saudi context, multiple studies have identified limited awareness of thyroid conditions among diverse populations, particularly among youth and nonhealth majors [8]. Educational campaigns targeting these demographics can potentially enhance early recognition, diagnosis, and treatment of hypothyroidism, thereby reducing long‐term complications [9].

Despite this growing body of Saudi literature, little attention has been given to hypothyroidism awareness among female health sciences students. This subgroup is uniquely important because these students represent future healthcare professionals who will play a central role in patient education, early recognition of thyroid disorders, and community health promotion. To the best of our knowledge, this is the first study in Saudi Arabia to specifically examine knowledge of hypothyroidism within this population. By focusing on an academically health‐oriented cohort, the study extends existing research by assessing whether future health providers possess adequate foundational understanding of a highly prevalent endocrine condition. For that reason, the aim of this study was to evaluate the extent of knowledge and awareness regarding hypothyroidism among female university students.

## 2. Significance of the Study

Hypothyroidism represents a significant global health burden, with well‐established epidemiological disparities across regions. In developed nations, the condition’s prevalence in the general population is consistently estimated at approximately 4%‐5% [10]. This figure contrasts sharply with a reported rate of 47.34% from a study conducted in the Hail region of Saudi Arabia [11]. To accurately interpret this divergence, critical contextualization is required. The elevated figure from Saudi Arabia is derived from a clinic‐based sample, reflecting the pattern of thyroid disease among patient attendees within a specific regional healthcare setting, rather than representing a general population prevalence. This distinction is crucial to avoid misinterpretation, as it highlights the difference between community‐wide epidemiological data and findings from clinical or regional cohorts, which may be influenced by healthcare‐seeking behavior and local diagnostic practices. Although hypothyroidism is relatively common, awareness of the condition especially among young Saudi women remains limited. This study therefore seeks to address existing knowledge gaps by evaluating the level of understanding of hypothyroidism among female health science college students. Identifying specific areas of insufficient awareness will support the design of targeted educational strategies aimed at strengthening thyroid‐related health literacy. Enhancing young women’s access to accurate information about hypothyroidism not only contributes to their personal health but also promotes broader community well‐being by enabling more informed health‐related choices in the future.

## 3. Subject and Methods

### 3.1. Study Design

This study employed a descriptive cross‐sectional approach.

### 3.2. Research Questions

The study was guided by the following research questions:

1. What is the level of knowledge about hypothyroidism among female university students?
2. How aware are these students of the symptoms, causes, and implications of hypothyroidism?
3. Is there a statistically significant relationship between the level of knowledge about hypothyroidism and selected demographic variables such as age, academic level, field of study, and personal or family history of thyroid disorders among female students?

### 3.3. Study Setting

The study was carried out at the female campus of Algouf University in Saudi Arabia, targeting students enrolled in health sciences programs. Participants were drawn from three major specialties Nursing, Medical Laboratory Sciences, and Physiotherapy. Data collection spanned a four‐month period, from September through December 2024, ensuring coverage across an entire academic semester and allowing for broad representation of student experiences within these disciplines.

### 3.4. Study Population and Sampling Strategy

A convenience sampling technique was employed to recruit eligible participants for the study. In total, 384 female students aged 18 years and above, representing various academic programs and levels of study, voluntarily participated. Postgraduate students and students with a previously diagnosed thyroid disorder were excluded to avoid bias in knowledge assessment. The sample included students from three major disciplines: 150 from the College of Nursing, 180 from the Medical Laboratory program, and 80 from Physiotherapy. Based on the number of students enrolled in these programs, the study achieved a high overall response rate of 93.6%, indicating strong participation and engagement from the targeted population.

### 3.5. Sample Size Determination

The sample size was calculated using the formula for estimating a proportion in a population. *n* = Z^2^⋅*p*⋅(1−*p*)/*d* ^2^where *n* = required sample size, Z = Z‐value (1.96 for 95% confidence level), *p* = estimated prevalence of adequate knowledge (assumed at 50% for maximum variability), and *d* = margin of error (0.05). The minimum required sample size was 384.

### 3.6. Data Collection Tools

One tool was used to collect the data, namely: A structured, self‐administered electronic questionnaire was developed for data collection. The tool was designed based on an extensive review of recent literature [2, 6, 12, 13] and subjected to expert evaluation and pilot testing. Knowledge about hypothyroidism was assessed using a structured questionnaire composed of 37 dichotomous items, with each item scored as 1 for a correct response and 0 for an incorrect, “no,” or “I don’t know” response. The questionnaire was organized into 11 knowledge domains, each representing a distinct conceptual area relevant to thyroid health. These domains included position and anatomy of the thyroid gland (4 items), function of the thyroid gland (2 items), hormones of the thyroid gland (3 items), terminologies related to thyroid disorders (5 items), definition of hypothyroidism (2 items), causes of hypothyroidism (2 items), signs and symptoms of hypothyroidism (4 items), complications of hypothyroidism (4 items), treatment of hypothyroidism (2 items), common misconceptions (7 items), and awareness regarding thyroid screening (3 items). Domain scores therefore ranged from 0 to the number of items within each domain, and results are presented as the number and percentage of participants who answered each domain correctly or incorrectly. A total knowledge score was calculated by summing all 37 items and subsequently converting the score into a percentage. Based on established educational performance standards, overall knowledge was categorized into poor (< 65%), moderate (65% to < 70%), and high (≥ 70%) levels, with higher scores indicating better knowledge.

### 3.7. Validity and Reliability

To ensure content and face validity, the questionnaire underwent a thorough evaluation by five pediatric nursing experts. Subsequently, a pilot study involving 10% of the calculated sample (excluded from the final analysis) was conducted to assess clarity, feasibility, and time requirements. Based on feedback, necessary amendments were implemented. The internal consistency reliability of the final instrument was confirmed with Cronbach’s alpha of 0.87, indicating strong reliability.

### 3.8. Data Collection Procedures

Researchers introduced the study to participants, explained its objectives, and distributed the questionnaire both in person and electronically via Google Forms and WhatsApp to maximize participation.

### 3.9. Ethical Considerations

All study procedures were conducted in strict accordance with established ethical standards and the principles outlined in the Declaration of Helsinki. Ethical approval was granted by the Research Ethics Committee at the Faculty of Nursing, Mansoura University (IRB No. 0851). Participation was entirely voluntary, and informed consent was obtained electronically prior to data collection. The Google Form used for the survey included a mandatory consent checkbox: participants who selected “Yes” were directed to the questionnaire, while those who selected “No” were automatically exited from the survey, thereby ensuring written consent was secured before participation. To facilitate broad and accessible recruitment, the study was disseminated through social media platforms such as WhatsApp and university‐affiliated groups, specifically targeting female postgraduate students across different academic levels. The online questionnaire provided a clear explanation of the study’s objectives, assurances of confidentiality, and a detailed consent statement. No personal identifiable information was collected, and all responses were anonymized to safeguard participant privacy and data security.

### 3.10. Data Analysis and Statistical Tests

Data were analyzed using the Statistical Package for the Social Sciences (SPSS) software, version 22 (IBM SPSS Statistics for Windows, Version 22.0). Categorical variables were presented using frequencies and percentages, while continuous variables were summarized using means and standard deviations. The Chi‐square test was employed to evaluate associations between different variables. A *p* value of less than 0.05 was considered to indicate statistical significance. Before conducting multivariable analysis, multicollinearity was assessed. All VIF values were < 2.5, indicating no multicollinearity concerns. The Test of Parallel Lines was nonsignificant (*χ* ^2^ = 12.47, df = 8, *p* = 0.131), confirming that the proportional odds assumption was met, justifying the use of ordinal logistic regression.

## 4. Results

A total of 384 female students participated in the study. The majority (45.8%) were between the ages of 20 and 23 years, with a mean age of 21.6 ± 2.4 years. Most participants were enrolled in the fifth or sixth academic levels (34.6%), and nearly half (47.1%) were from the nursing program. Additionally, 59.4% of the students reported having a personal or family history of thyroid disorders (Table 1).

**TABLE 1:** Demographic characteristics of the studied participant (*n* = 384).

Items	*n* (384)	%
Age 18–< 20 y	49	12.8

20 < 23 y	176	45.8

> 23 y	159	41.4

Mean age ± SD	21.6 ± 2.4	

University level first and second	43	11.2

Third and fourth	84	21.9

Fifth and sixth	133	34.6

Seventh and eighth	124	32.3

Specialty laboratories	103	26.8

Nursing	181	47.1

Physiotherapy	100	26.1

Personal and family history related to thyroid disorders Yes	228	59.4

No	156	40.6

Participants’ knowledge levels varied across the 11 domains assessed by the 37‐item questionnaire. Table 2 presents the distribution of correct and incorrect responses for each domain. The highest proportion of correct responses was observed in the domain of terminologies associated with thyroid disorders (75.0%), followed by position and anatomy of the thyroid gland (70.8%) and treatment of hypothyroidism (69.0%). The lowest rates of correct answers were observed in identifying the definition of hypothyroidism (60.9%) and in awareness of thyroid screening (60.9%), indicating that knowledge levels varied considerably across different aspects of hypothyroidism.

**TABLE 2:** Distribution of studied subjects’ knowledge regarding hypothyroidism (*n* = 384).

Knowledge items	Item (*n*)	Correct		Incorrect	
		*n*	%	*n*	%
Position and anatomy of the thyroid glands	4	272	70.8	112	29.2
Function of thyroid glands	2	249	64.8	135	35.2
Hormones of thyroid glands	3	255	66.4	129	33.6
Terminologies associated with thyroid disorders	5	288	75	96	25
Definition of hypothyroidism	2	234	60.9	150	39.1
Causes of hypothyroidism	2	250	65.1	134	34.9
Signs and symptoms related to hypothyroidism	4	250	65.1	134	34.9
Complications of hypothyroidism	4	254	66.1	130	33.9
Treatment of hypothyroidism	2	265	69.0	119	31.0
Common misconceptions regarding thyroid disorders	7	253	65.8	131	34.2
Awareness regarding thyroid screening	3	234	60.9	150	39.1

**FIGURE 1:**
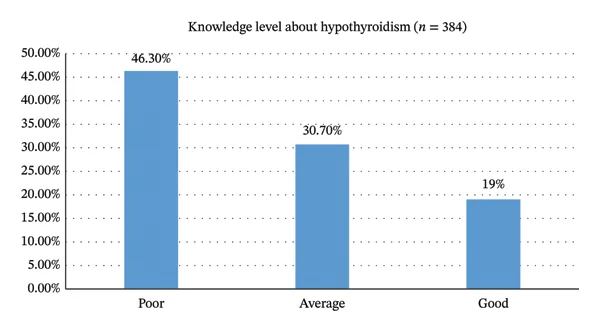
Distribution of knowledge levels on hypothyroidism among female students.

Figure 1 indicates that approximately 46.3% of the respondents were classified as having low knowledge, pointing to a significant gap in understanding, even among students likely enrolled in health‐related programs. In contrast, only 19% showed a high level of knowledge, which may reflect shortcomings in the academic curriculum or a general lack of awareness about hypothyroidism.

Statistical analysis revealed significant associations between knowledge levels and several demographic variables (Table 3). Students over the age of 23 were more likely to have high knowledge (53.4%) compared to younger age groups (*p* < 0.001). Similarly, students in the seventh and eighth academic levels exhibited higher knowledge scores (50.7%) than those in earlier levels (*p* < 0.001). Nursing students had the highest proportion of high knowledge (56.2%), followed by laboratory and physiotherapy students. Furthermore, students with a personal or family history of thyroid disorders were significantly more knowledgeable, with 75.9% falling into the moderate or high knowledge categories (*p* < 0.001).

**TABLE 3:** Relationship between characteristics of studied subjects and their knowledge regarding hypothyroidism (*n* = 384).

Characteristics	Knowledge levels						Test of significance	
	Poor		Moderate		High		*χ* ^2^	*p*
	No.	%	No.	%	No.	%		
Age 18 < 20 y	11	6.2	25	18.8	13	17.8	54.29	< 0.001^∗^
20 < 23 y	118	66.3	37	27.8	21	28.8		
> 23 y	49	27.5	71	53.4	39	53.4		

University level first and second	28	15.7	11	8.3	4	5.5	66.47	< 0.001^∗^
Third and fourth	43	24.2	28	21	13	17.8		
Fifth and sixth	85	47.8	29	21.8	19	26		
Seventh and eighth	22	12.3	65	48.9	37	50.7		

Specialty laboratories	47	26.4	36	27.1	20	27.4	48.67	< 0.001^∗^
Nursing	67	37.6	73	54.9	41	56.2		
Physiotherapy	64	36	24	18	12	16.4		

Personal and family history related to thyroid disorders Yes	76	42.7	101	75.9	51	69.9	61.19	< 0.001^∗^
No	102	57.3	32	24.1	22	30.1		

^∗^Statistically significant at *p* ≤ 0.05.

Multivariable ordinal logistic regression was performed (Table 4) to identify independent predictors of knowledge levels (poor, moderate, and high as ordered categories). The proportional odds (parallel lines) assumption was tested using the Test of Parallel Lines; a nonsignificant result (*p* > 0.05) indicated the assumption was met. Variables with *p* < 0.20 in bivariate analysis or those deemed clinically important were entered into the multivariable model. Multicollinearity was assessed using the variance inflation factor (VIF), with VIF < 5 considered acceptable. Model fit was evaluated using the deviance goodness‐of‐fit test, Akaike Information Criterion (AIC), Bayesian Information Criterion (BIC), and pseudo*R* ^2^ (Nagelkerke). Adjusted odds ratios (AOR) with 95% confidence intervals (CI) were calculated. The ordinal logistic regression model demonstrated acceptable fit and predictive performance. The overall model was statistically significant (*χ* ^2^ = 142.35, df = 8, *p* < 0.001), with −2 Log Likelihood = 687.24. Pseudo *R* ^2^ values indicated moderate explanatory power (Cox and Snell *R* ^2^ = 0.304; Nagelkerke *R* ^2^ = 0.341; McFadden *R* ^2^ = 0.171). Information criteria were AIC = 711.24 and BIC = 762.18. Goodness‐of‐fit tests supported model adequacy, with deviance *χ* ^2^ = 647.82 (df = 698, *p* = 0.912) and Pearson *χ* ^2^ = 721.45 (df = 698, *p* = 0.258), both nonsignificant, indicating good fit. The assumption of proportional odds was met (Test of Parallel Lines: *χ* ^2^ = 12.47, df = 8, *p* = 0.131). Overall classification accuracy was 58.3%, with the category‐specific accuracy of 62.4% for “Poor,” 51.9% for “Moderate,” and 57.5% for “High.” The regression analysis output included the estimated coefficients (B), their corresponding standard errors (SE), AOR, 95% CI, and the VIF used to assess multicollinearity. Variables marked with an asterisk (∗) were statistically significant at *p* ≤ 0.05.

**TABLE 4:** Multivariable ordinal logistic regression analysis of independent predictors of higher knowledge levels regarding hypothyroidism (*n* = 384).

Predictor variable	B	SE	Wald *χ* ^2^	df	*p*	AOR	95% CI	VIF
Age group			18.42	2	< 0.001^∗^			
18–< 20 y	—	—	—	—	—	1.00 (Ref)	—	—
20–< 23 y	0.42	0.34	1.51	1	0.218	1.52	0.78–2.96	2.14
> 23 y	1.35	0.35	14.73	1	< 0.001^∗^	3.84	1.92–7.68	2.38
University level			21.65	3	< 0.001^∗^			
First and second	—	—	—	—	—	1.00 (Ref)	—	—
Third and fourth	0.64	0.38	2.73	1	0.098	1.89	0.89–4.02	1.87
Fifth and sixth	0.55	0.37	2.20	1	0.139	1.73	0.84–3.57	2.05
Seventh and eighth	1.74	0.39	19.85	1	< 0.001^∗^	5.67	2.65–12.13	2.21
Specialty			13.28	2	0.001^∗^			
Physiotherapy	—	—	—	—	—	1.00 (Ref)	—	—
Laboratories	0.52	0.28	3.41	1	0.065	1.68	0.97–2.91	1.45
Nursing	1.08	0.27	15.74	1	< 0.001^∗^	2.94	1.72–5.03	1.52
Personal/family history of thyroid disorders			31.84	1	< 0.001^∗^			
No	—	—	—	—	—	1.00 (Ref)	—	—
Yes	1.31	0.21	39.42	1	< 0.001^∗^	3.72	2.45–5.65	1.18

^∗^Statistically significant at *p* ≤ 0.05.

## 5. Discussion

Thyroid disorders represent some of the most prevalent chronic health conditions worldwide. The thyroid gland, an essential organ within the endocrine system, secretes hormones that regulate physiological functions across nearly all body systems. Among thyroid‐related conditions, hypothyroidism and hyperthyroidism occur most frequently. Hypothyroidism is characterized by clinical and biochemical deficiency in thyroid hormone production. Although commonly encountered and typically manageable, inadequate treatment can lead to severe and potentially life‐threatening complications [14]. The present study aims to assess health science college students’ awareness and understanding of hypothyroidism. In the context of Saudi Arabia, rapid socioeconomic development and lifestyle transitions associated with urbanization have shaped public health practices and levels of health awareness, underscoring the need for updated and targeted health education initiatives.

The findings of this study indicate a substantial gap in hypothyroidism‐related knowledge among students in health‐related disciplines, with 46.3% demonstrating low levels of understanding. This is particularly concerning given their anticipated responsibilities as future healthcare professionals. Comparable patterns have been documented in previous research. For example, a study among paramedical students reported that nearly one‐third had inadequate knowledge and over one‐fifth showed poor awareness of hypothyroidism, highlighting the need for more comprehensive educational initiatives [15]. Similarly, research involving healthcare students in Saudi Arabia identified limited awareness of thyroid cancer especially regarding its risk factors and early warning signs underscoring the importance of strengthening endocrine‐related educational strategies [13].

The findings of this study indicate a notable deficiency in hypothyroidism‐related knowledge among health science students. We speculate that this pattern may be related to the structure of early academic curricula, in which emphasis is typically placed on general medical sciences, while more specialized clinical topics such as hypothyroidism are introduced later or addressed only briefly. This might also reflect limited opportunities for clinical exposure or interactive, case‐based learning, which have been shown in other educational contexts to enhance retention and application of theoretical concepts. Additionally, the study observed that students with a personal or family history of thyroid disease demonstrated higher knowledge levels, suggesting that prior exposure may contribute to greater awareness. This aligns with earlier research reporting an 8% prevalence of hypothyroidism among college students, particularly older females, and noting that familiarity with hypothyroidism within the family is associated with increased understanding [16, 17]. The observed relationship between advanced academic standing and greater knowledge highlights the crucial role of the educational curriculum. Introducing thorough instruction on hypothyroidism early in health sciences programs could play a key role in addressing these knowledge deficiencies.

Therefore, the current study’s findings that reveal the stronger knowledge among students with a personal or family history of hypothyroidism may stem from direct exposure, which enhances awareness and motivation to learn. Similarly, students in advanced academic levels benefit from broader curricular content and clinical experience, reinforcing their understanding. These findings emphasize the impact of both experiential learning and structured education on health knowledge.

### 5.1. Limitations

Although this study offers valuable insights, several limitations should be acknowledged. The reliance on a self‐administered questionnaire may have introduced response bias, as participants could unintentionally misjudge their level of knowledge. Additionally, the cross‐sectional nature of the research captures information at only one moment in time, preventing conclusions about causality or changes in knowledge as students’ progress through their academic programs. The use of convenience sampling, while feasible, also restricts the generalizability of the results because the sample may not accurately reflect the wider university student population or students in similar programs elsewhere. Furthermore, the voluntary nature of participation, particularly through online recruitment raises the possibility of selection bias, as students with a stronger interest in health topics or higher motivation may have been more likely to participate. Future research would benefit from employing randomized sampling strategies and longitudinal designs to strengthen the validity and broader applicability of the findings.

## 6. Conclusion and Recommendations

The findings of this study demonstrate a considerable gap in knowledge and awareness of hypothyroidism among female students in health‐related disciplines, with nearly half of the participants exhibiting low levels of understanding despite the clinical relevance of the condition. Higher knowledge scores were more common among older students, those in advanced academic years, nursing students, and individuals with a personal or family history of hypothyroidism. These results highlight the importance of strengthening thyroid‐related education within health sciences programs, particularly for students in the early stages of their training. Recommended strategies include integrating focused thyroid health content into undergraduate coursework, adopting interactive instructional approaches such as case‐based learning, and implementing faculty‐led awareness initiatives within health colleges. Additional efforts such as peer‐led education, collaboration with clinical specialists, and the use of digital platforms for health promotion may further enhance students’ understanding.

While the present study did not include students from nonhealth majors, future research should expand to these groups, who may have even lower levels of thyroid‐related awareness. Broader university‐wide educational campaigns could also be explored as part of future practice implications. Longitudinal studies and comparative evaluations of different teaching methods are recommended to better assess the long‐term impact of educational interventions and to inform the development of more effective thyroid health education strategies.

## Funding

No funding was received for this manuscript.

## Conflicts of Interest

The authors declare no conflicts of interest.

## Data Availability

Data sharing is not applicable to this article as no datasets were generated or analyzed during the current study.
